# A rare case of mucormycosis in a diabetic patient: diagnostic challenges and clinical management of mucormycosis hand infection

**DOI:** 10.1080/23320885.2024.2333879

**Published:** 2024-03-31

**Authors:** Hatan Mortada, Razan Albrahim, Saad Alrobaiea, Moinuddin Ahmad, Elfayad Hamad A. Abdelraheem, Muath Hakami

**Affiliations:** a Division of Plastic Surgery, Department of Surgery, King Saud University Medical City, King Saud University, Riyadh, Saudi Arabia; b Department of Plastic Surgery and Burn, King Saud Medical City, King Saud University, Riyadh, Saudi Arabia; c College of Medicine and Surgery, Princess Noura Bin Abdulrahman University, Riyadh, Saudi Arabia; d Department of Plastic Surgery and Burn Unit, Security Forces Hospital, Riyadh, Saudi Arabia; e Division of Plastic Surgery, Department of Surgery, King Fahad Medical City, Riyadh, Saudi Arabia

**Keywords:** Mucormycosis, zygomycosis, diabetes mellitus, hand, cutaneous, fungal infection, Immunosuppression

## Abstract

Mucormycosis hand infection in poorly controlled diabetic presented as rapidly progressive swelling, redness, pain, and necrosis unresponsive to antibiotics. Prompt diagnosis and aggressive surgery, antifungals, and diabetes management were critical, highlighting the need for early recognition and treatment of mucormycosis in diabetics.

## Introduction

Mucormycosis, also known as zygomycosis, is an opportunistic fungal infection with an increasing incidence globally [1–3]. It has frequently been observed in individuals with diabetes mellitus, hematological malignancies, organ transplantations, corticosteroid use, history of trauma, and following burns [3,4]. The most prevalent manifestations include rhinocerebral, cutaneous, and pulmonary mucormycosis, while less common forms like gastrointestinal and disseminated forms have been described [2]. We report here on this rare case in a diabetic patient, shedding light on a potentially lethal infection if detected late. Additionally, we present a comprehensive review of the clinical and surgical features of similar cases reported in the literature.

## Case study

A 74-year-old unemployed, male with a known history of diabetes mellitus, hypertension, trigeminal neuralgia, and prostate cancer presented to our emergency department (ED) complaining of severe pain and swelling of the ring and little fingers of the left hand.

The patient had a history of falling on his left hand, resulting in a fracture of the distal phalanx of the little finger three weeks prior. The fracture was treated conservatively with a cast at another institute. One week later, he developed blistering and an abscess over the volar base of the left little finger, which was managed by incision and drainage. The pain persisted and his condition progressively worsened until he presented to our ED.

**Figure 1.**
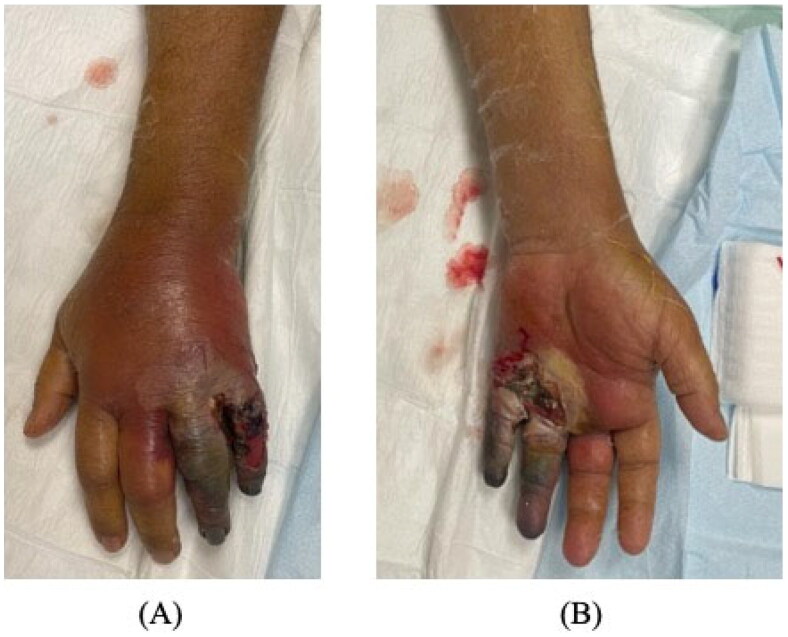
Wet gangrene of the little and ring fingers on initial presentation, with diffuse swelling reaching the palm. (A) Dorsal. (B) Volar.

On examination, the patient was alert and oriented with stable vital signs (blood pressure 186/86 mmHg, pulse 95 beats/min, respiratory rate 20 breaths/min, temperature 36.9 °C, and oxygen saturation 94% on room air). Local examination demonstrated left hand wet gangrene of the ring and little fingers with diffuse swelling reaching the palmar surface and middle finger. The surrounding region was warm, tender with marked erythema (Figure 1). There were no crepitations or fluctuations.

**Figure 2.**
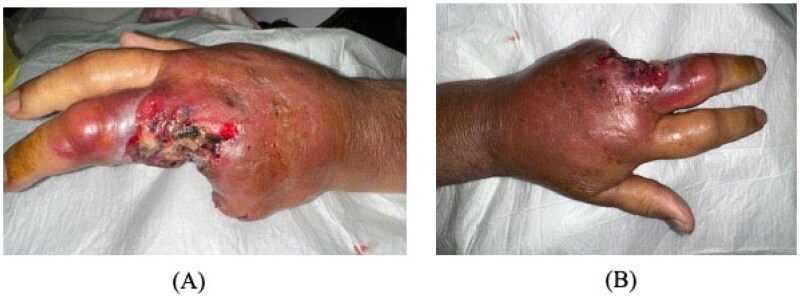
First day after ray amputation of the ring and little fingers. Persistent swelling and erythema involving the middle finger with no improvement of palm swelling and erythema on antibiotics.

Laboratory findings showed a white blood count of 12.6 × 10^9^/L, hemoglobin 11.4 g/dL, C-reactive protein 247 mg/dL, and HbA1c 7.19%. X-ray of the hand revealed soft tissue swelling and calcifications around the fourth and fifth fingers. (Figure 2)

**Figure 3.**
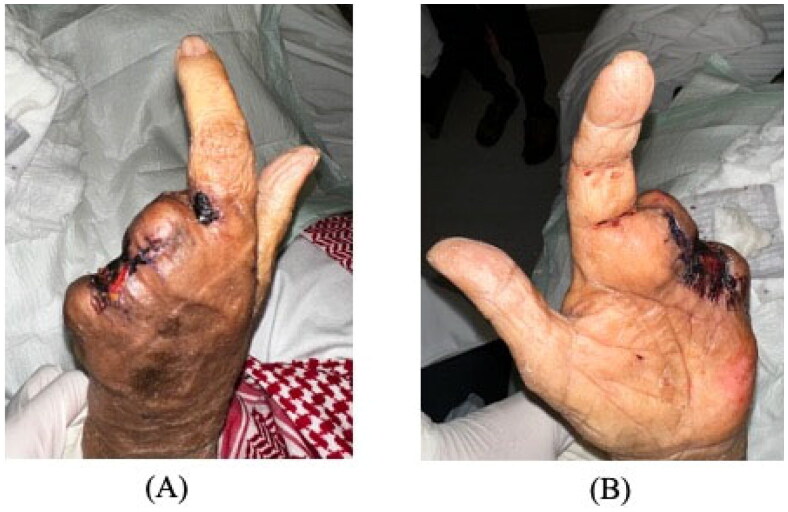
Ten days after middle finger amputation, prior to discharge. Marked improvement of swelling and erythema in the palm is seen after starting antifungal therapy.

The patient was admitted and started on vancomycin and tazocin with strict blood glucose and blood pressure control. He underwent ray amputation of the ring and little fingers, amputation stump was left open with regular dressing changes in the early postoperative period due to the high risk of ongoing infection. Initial wound culture was negative for bacteria, as were blood and urine cultures. MRSA screening was also negative (Figure 2). On day 6, tissue culture showed zygomycetes growth. The patient was immediately started on intravenous amphotericin B 400 mg daily. Over the following days, the erythema and swelling decreased without signs of infection. Left middle finger amputation was ultimately performed on day 25 due to progressive skin necrosis and decreased vascularity. Over the following days, the erythema and swelling decreased without signs of infection. Later on, the patient was discharged with a complete resolution of symptoms (Figure 3). The patient was followed up in the outpatient department at a 1-month, and 3-month intervals with no evidence of recurrence or fungal infection elsewhere.

## Discussion

Cutaneous mucormycosis primarily affects immunocompromised patients, especially those with severe neutropenia (less than 500 neutrophils/mm^3^), hematologic malignancies, and diabetic ketoacidosis [5]. Although HIV/AIDS is a known risk factor for fungal infections, it does not independently increase mucormycosis risk. This is likely because neutrophils, which are unaffected by HIV, are the main defense against Mucor. Instead, intravenous drug use has been proposed as the most probable cause of mucormycosis infection in HIV/AIDS patients [6]. Overall, mucormycosis largely remains a disease of profound immunodeficiency, particularly neutropenia, hematologic malignancies, and uncontrolled diabetic ketoacidosis rather than HIV/AIDS alone [5,6]. In this case report, we present a unique case of a diabetic patient with mucormycosis infection of the hand. Initially suspected to be a bacterial infection, the patient did not improve on antibiotics. The main aim is to increase awareness about such atypical presentations of mucormycosis and to consider fungal infections when not responding to antibiotics, even in immunocompetent diabetic patients. This case highlights the importance of having a high index of suspicion for mucormycosis, as early diagnosis and treatment are crucial.

In immunocompetent patients, trauma is the risk factor most strongly associated with developing cutaneous mucormycosis, as seen in this case where the patient had suffered a fracture and was placed in a splint prior to developing the infection [5]. In our review, we found only one reported case of cutaneous mucormycosis occurring in an immunocompetent patient without previous trauma [7]. In all other reported cases, the trauma preceding mucormycosis ranged from major crush injuries with significant soil contamination to minor intramuscular injections. The trauma provides an entry point for the fungi to inoculate the tissue and establish infection [8].

The typical clinical course of mucormycosis and the devastating consequences of delayed treatment have been well described. The initial factor is damage to the skin allowing fungal inoculation. Early signs include pustules, blisters, nodules, ulcerations, gangrenous lesions, and necrotizing cellulitis [3,4,9]. The temporal progression can be acute, mimicking necrotizing fascitis, or more indolent over years. As Al-Qattan et al. described, the infection often takes an acute course, and could be divided into three clinical stages. The first concerning sign is an enlarging black eschar at the wound site. This is followed by bleeding from the subcutaneous vessels, ultimately progressing into a digital gangrene. Without treatment, the infection disseminates into the subcutaneous vasculature and eventually major limb arteries. This predicts poor outcomes including likely amputation and potential widespread dissemination [10]. Mucormycosis follows a rapidly progressive course with high morbidity if diagnosis and treatment are delayed. Careful inspection for eschar formation and having a high index of suspicion are critical for early detection and intervention [5,6].

Extensive surgical debridement remains the primary treatment for mucormycosis, with antifungal therapy playing an adjuvant role [10]. Debridement must remove all necrotic tissue along with margins of uninfected tissue. Any further spread necessitates repeat debridement until no signs of infection remain and all eschar is gone. If multiple debridements fail to control the infection, amputation should be considered to prevent dissemination. The mainstay of antifungal therapy is amphotericin B, which risks renal damage, hypokalemia, and infusion reactions [2–5]. Following amputation, open wound care allows for direct visualization and monitoring of the wound bed, facilitating infection control and tissue debridement. Studies suggest its benefits in promoting granulation tissue formation and reducing infection rates [11]. Primary closure of amputation is safe if infection control is achieved, and evidence of healthy tissue is present [12]. Hyperbaric oxygen therapy shows promise as an adjunctive treatment for cutaneous zygomycosis, especially in diabetics. It involves breathing pure oxygen in a pressurized chamber, promoting wound healing, and inhibiting fungal growth [13].

Liposomal formulations allow higher doses with fewer side effects, but are more expensive and could reach up to a total of 55,324 $ when combined with hospital length of stay, complications, and treatment outcomes [14]. This report is limited by several factors. As the initial hand injury was treated at an outside facility, images and details from the first admission are not available. Additionally, we were unable to obtain MRI imaging to fully characterize the extent of soft tissue involvement. Longer-term postoperative follow-up is lacking given challenges with the patient traveling from out of town for in-person visits. However, phone follow-up at 3 months did confirm he was doing well with no signs of recurrence. Finally, histopathology images demonstrating characteristic broad, ribbon-like mucormycete hyphae were unable to be obtained but would have added value in supporting the diagnosis. Despite these limitations, this case aims to highlight an exceptionally rare and dangerous infection in the setting of poorly controlled diabetes. In summary, early and aggressive surgical debridement is critical, coupled with antifungal therapy using liposomal amphotericin B when feasible. Hyperbaric oxygen may provide adjunctive benefit. A multimodal approach is required for successful treatment.

## Conclusion

Cutaneous mucormycosis is a serious fungal infection that can be fatal if not treated expeditiously. A high index of suspicion should be maintained in patients with the aforementioned risk factors, as well as those who do not respond to empiric antibiotics. Prompt diagnosis, timely surgical debridement, and commencement of appropriate antifungal therapy are essential for management and improved outcomes.

## References

[1] Skiada A, Drogari-Apiranthitou M, Pavleas I, et al. Global cutaneous mucormycosis: a systematic review. J Fungi (Basel). 2022;8(2):1. doi: 10.3390/jof8020194. PMC8878367 35205948

[2] Castrejón-Pérez AD, Welsh EC, Miranda I, et al. Cutaneous mucormycosis. An Bras Dermatol. 2017;92(3):304–5. doi: 10.1590/abd1806-4841.20176614. 29186239 PMC5514567

[3] Pal M. Zygomycosis: a highly infectious emerging opportunistic fungal disease of public health concern. OAJMMS. 2020;3(2). doi: 10.23880/OAJMMS-16000122.

[4] Jeong W, Keighley C, Wolfe R, et al. The epidemiology and clinical manifestations of mucormycosis: a systematic review and meta-analysis of case reports. Clin Microbiol Infect. 2019;25(1):26–34. PMID: 30036666. doi: 10.1016/j.cmi.2018.07.011. 30036666

[5] Spellberg B, Edwards J, Ibrahim A. Novel perspectives on mucormycosis: pathophysiology, presentation, and management. Clin Microbiol Rev. 2005;18(3):556–569. doi: 10.1128/CMR.18.3.556-569.2005. 16020690 PMC1195964

[6] Lanternier F, Dannaoui E, Morizot G, et al. A global analysis of mucormycosis in France: the RetroZygo study (2005–2007). Clin Infect Dis. 2012;54 (suppl 1):S35–S43. doi: 10.1093/cid/cir866. 22247443

[7] Mantadakis E, Samonis G. Clinical presentation of zygomycosis. Clin Microbiol Infect. 2009;15(5):15–20. doi: 10.1111/j.1469-0691.2009.02978.x. 19754751

[8] Tiong WHC, Ismael T, McCann J. Post-traumatic and post-surgical absidia corymbifera infection in a young, healthy man. J Plast Reconstr Aesthet Surg. 2006;59(12):1367–1371. doi: 10.1016/j.bjps.2006.03.044. 17113521

[9] Roden MM, Zaoutis TE, Buchanan WL, et al. Epidemiology and outcome of zygomycosis: a review of 929 reported cases. Clin Infect Dis. 2005;41(5):634–653. doi: 10.1086/432579. 16080086

[10] Al-Qattan MM, Al Mazrou AM. Mucormycosis of the upper limb. J Hand Surg Br. 1996;21(2):261–262. doi: 10.1016/s0266-7681(96)80111-2.PMID: 8732414. 8732414

[11] Ali Y, Halvorson J, Nunn A, et al. Delayed closure is associated with decreased infection rate in amputations after trauma. Am Surg. 2019;85(5):501–504. PMID: 31126363. 31126363

[12] Lakstein D, Feldbrin Z, Schorr L, et al. Primary closure of elective toe amputations in the diabetic foot–is it safe? J Am Podiatr Med Assoc. 2014;104(4):383–386. PMID: 25076082. doi: 10.7547/0003-0538-104.4.383. 25076082

[13] Bentur Y, Shupak A, Ramon Y, et al. Hyperbaric oxygen therapy for cutaneous/soft-tissue zygomycosis complicating diabetes mellitus. Plast Reconstr Surg. 1998;102(3):822–824. PMID: 9727450. doi: 10.1097/00006534-199809030-00030. 9727450

[14] Mistro S, Gomes B, Rosa L, et al. Cost-effectiveness of liposomal amphotericin B in hospitalised patients with mucocutaneous leishmaniasis. Trop Med Int Health. 2017;22(12):1569–1578. doi: 10.1111/tmi.12996. Epub 2017 Nov 22. PMID: 29078022. 29078022

